# Antitumor Efficacy of EGFR-Targeted Recombinant Immunotoxin in Human Head and Neck Squamous Cell Carcinoma

**DOI:** 10.3390/biology11040486

**Published:** 2022-03-22

**Authors:** Guiqin Xie, Liang Shan, Yuanyi Liu, Tzyy-Choou Wu, Xinbin Gu

**Affiliations:** 1 Department of Oral Pathology and Cancer Center, Howard University, Washington, DC 20059, USA; 2 Cancer Center, Howard University, Washington, DC 20059, USA; shanliang1964@gmail.com; 3 Angimmune LLC, Rockville, MD 20855, USA; yyliu6@gmail.com; 4 Pathology, Oncology, Obstetrics & Gynecology and Molecular Microbiology &Immunology, Johns Hopkins University School of Medicine, Baltimore, MD 21287, USA; wutc@jhmi.edu

**Keywords:** head and neck squamous cell carcinoma, HNSCC, EGFR, recombinant immunotoxin, efficacy

## Abstract

**Simple Summary:**

Head and neck squamous cell carcinoma (HNSCC) is the sixth most common cancer worldwide, with more than 500,000 new cases diagnosed annually. Surgical resection, chemoradiotherapy, targeted therapy, and immunotherapy have been approved for HNSCC treatment. While a minority of patients experience dramatic long-lasting and favorable clinical responses, the majority of patients fail to achieve durable clinical responses. Thus, alternative options with improved beneficial response are urgently needed. In HNSCC, over 90% of tumors overexpress the cell surface epidermal growth factor receptor (EGFR). We previously produced a humanized recombinant immunotoxin, hDT806, targeting tumor-specific overexpressed EGFR and/or the EGFRvIII mutant. Here, we set out to explore the effects and mechanisms of hDT806 in treating HNSCC in both in vitro and in vivo settings. We found that hDT806 exhibits a significant cytotoxicity in HNSCC through disrupting EGFR signaling, transcription inhibition, and inducing apoptosis and DNA damage.

**Abstract:**

Over 90% of head and neck squamous cell carcinoma (HNSCC) overexpresses the epidermal growth factor receptor (EGFR). However, the EGFR-targeted monotherapy response rate only achieves 10–30% in HNSCC. Recombinant immunotoxin (RIT) often consists of an antibody targeting a tumor antigen and a toxin (e.g., diphtheria toxin [DT]) that kills cancer cells. We produced a humanized RIT, designated as hDT806, targeting overexpressed EGFR and investigated its effects in HNSCC. Distinct from the EGFR-targeted tyrosine kinase inhibitor erlotinib or antibody cetuximab, hDT806 effectively suppressed cell proliferation in the four HNSCC lines tested (JHU-011, -013, -022, and -029). In JHU-029 mouse xenograft models, hDT806 substantially reduced tumor growth. hDT806 decreased EGFR protein levels and disrupted the EGFR signaling downstream effectors, including MAPK/ERK1/2 and AKT, while increased proapoptotic proteins, such as p53, caspase-9, caspase-3, and the cleaved PAPR. The hDT806-induced apoptosis of HNSCC cells was corroborated by flow cytometric analysis. Furthermore, hDT806 resulted in a drastic inhibition in RNA polymerase II carboxy-terminal domain phosphorylation critical for transcription and a significant increase in the γH2A.X level, a DNA damage marker. Thus, the direct disruption of EGFR signaling, transcription inhibition, DNA damage, as well as apoptosis induced by hDT806 may contribute to its antitumor efficacy in HNSCC.

## 1. Introduction

The predominant form of head and neck cancers develops from the mucosal epithelium in the oral cavity, pharynx, and larynx, which is known collectively as head and neck squamous cell carcinoma (HNSCC). Globally, HNSCC accounts for >500,000 new cases annually, while in the United States, ~37,000 new cases are diagnosed with HNSCC every year [1,2]. The treatment approach to HNSCC patients is generally multimodal, involving surgery, chemoradiotherapy, and immunotherapy. Despite the therapeutic advances that have emerged in recent years, the 5-year survival rate of patients with advanced HNSCC remains 55–65% [3], and the prognosis for patients with recurrent or metastatic HNSCC is even poorer, with a median overall survival <1 year [4], highlighting the importance of developing alternative treatment strategies for HNSCC. The epidermal growth factor receptor (EGFR), a member of the HER/ErbB family of the tyrosine kinase transmembrane receptors, regulates cellular activities including cell cycle progression, proliferation, anti-apoptosis, and migration. In HNSCC, oncogenic EGFR alteration is one of the most notable characteristics. About 90% of HNSCC tumors overexpress the EGFR and HNSCC patients with a high expression of the EGFR have been associated with reduced survival and poor prognosis [5,6]. However, the only FDA-approved EGFR-targeted monoclonal antibody, cetuximab, achieves a response rate only at 10–30% as a monotherapy in HNSCC [7]. The evidence indicates that although the EGFR may serve as a valid target of therapeutic interventions, there is an urgent need to develop novel EGFR-targeted therapies for better efficacy in HNSCC.

Recombinant immunotoxin (RIT) represents a promising therapeutic for cancer therapy. As a group of chimeric proteins, an RIT often comprises an antibody and a toxin moiety such as diphtheria toxin (DT), which can be engineered to target a tumor antigen and kill cancer cells. RITs have been approved for treating several types of hematopoietic malignancies [8,9,10,11,12]. However, RITs are unable to achieve a satisfactory efficacy against solid tumors due to difficulties including effectively delivering them into tumors and killing cancer cells without harming normal tissues. Preclinical DT-based therapy has been explored in various types of solid tumor, such as hepatocellular cell carcinoma [13], glioblastoma [14,15], breast cancer [16], and lung cancer [17] for its potent cytolethal effect. We previously developed a bispecific RIT targeting the overexpressed EGFR and EGFRvIII variant on cancer cells [15], designated as DT390-HuBiscFv806 (hDT806), by fusing two humanized single-chain variable fragments (scFv) derived from monoclonal antibody mAb806 to the truncated form of DT. This bivalent RIT hDT806 was designed to leverage the unique specificity of mAb806 to the open form of overexpressed EGFR and its mutated form, EGFRvIII [18,19]. The EGFR mutant EGFRvIII is a tumor-specific cell surface marker in malignant glioblastoma. In our published study [15], hDT806 showed a more remarkable inhibition in glioblastoma cells with EGFRvIII expression versus without in glioblastoma mouse xenograft models. hDT806 also showed cytotoxicity in a panel of HNSCC cells. However, the in vivo efficacy and the mechanisms underlying this cytotoxicity of hDT806 in HNSCC remain unknown.

In the current study, we assessed the efficacy of hDT806 to treat HNSCC and explored the mechanisms underlying the inhibition in cell proliferation and mouse xenograft tumor growth by hDT806. Our data indicate that HNSCC cells are highly sensitive to hDT806, while these cells have differential sensitivity to the anti-EGFR antibody cetuximab or the EGFR-specific tyrosine kinase inhibitor erlotinib. The treatment of hDT806 disrupted EGFR signaling and inhibited cellular transcription, leading to DNA damage, an apoptotic response, and tumor growth inhibition in HNSCC.

## 2. Materials and Methods

### 2.1. Cell Culture

The four HNSCC cell lines used in this study, including JHU-011 (p53 mutated), JHU-013 (p53 mutated), JHU-022 (wild type of p53), and JHU-029 (wild type of p53), were obtained from Johns Hopkins University [20,21,22]. These cells were cultured in Roswell Park Memorial Institute (RPMI) 1640 media (#21875034; Thermo Fisher Scientific, Waltham, MA, USA) with supplements of 10% fetal bovine serum (FBS; #16140071, Thermo Fisher Scientific) and 1% antibiotic-antimycotic solution (#15240062; Fisher Scientific) and incubated at 37 ℃ in a humidified incubator with 5% CO_2_.

### 2.2. Cell Viability Assay

The Crystal Violet Assay Kit (#ab232855; Abcam, Cambridge, UK) was used for cell cytotoxicity and cell viability studies, as previously described [22]. Briefly, the HNSCC cells were plated 3000–4000/well in 96-well plates and treated with hDT806, cetuximab (#A2000; Selleckchem, Houston, TX, USA), or erlotinib (#S1023; Selleckchem) following a 2-fold serial dilution. Five to seven days later, a crystal violet staining assay was performed according to the manufacturer’s instruction to determine cytotoxicity and cell viability. Optical density (O.D.) of each well was measured at 595 nm on a microplate reader. The percentage of viable (attached) cells against the values of untreated control samples were calculated to represent cell viability [23].

### 2.3. Flow Cytometry Apoptosis Assay

The FITC Annexin V Apoptosis Detection kit (#556547, BD Biosciences, Ann Arbor, MI USA) was used for apoptotic cell death assessment. Cells were treated with vehicle or hDT806 (20 nM) for 48 h, collected, and incubated with annexin V-FITC and propidium iodide (PI) solutions in the dark for 15 min. a flow cytometry assay was performed on a BD flowcytometer (BD Biosciences, San Jose, CA). Both the annexin V-positive and PI-negative cells and annexin V-positive and PI-positive cells were regarded as apoptotic cells. FlowJo software (FlowJo LLC, Ashland, OR) was used to analyze the percentage of apoptotic cells.

### 2.4. Western Blot Analysis

HNSCC cells cultured in six-well plates were treated with vehicle or hDT806 (20 nM) for 48 h, harvested, and washed with PBS. The collected cells were homogenized in a RIPA lysis buffer before centrifugation at 16,000× *g* for 20 min at 4 ℃, as previously described [22]. The protein concentrations of lysates were quantified, and 30 mg of lysates was used for Western blot analysis. The antibodies against EGFR (#4267s), *p*-AKT (#9271), total-AKT (#9272), *p*-ERK1/2 (#9101), total-ERK (#9102), poly (ADP ribose) polymerase 1 (PARP) (#9542), γH2A.X (#9718), H2A.X (#7631), RNAPII carboxy-terminal domain (CTD) *p*-Ser2/5 (#13546), *p*-Ser7 (#13780), and RNAPII large subunit Rpb1 (#2629) were purchased from Cell Signaling (Beverly, MA, USA). The antibody against β-actin (#47778 HRP) and ErbB2 (sc-284) were purchased from Santa Cruz (Dallas, TX, USA). The antibody against p53 (#OP03), caspase 3 (#c8487), and caspase 9 (#c7729) were purchased from Sigma-Aldrich (St. Louis, MO, USA). Anti-rabbit or anti–mouse IgG secondary antibodies conjugated with horseradish peroxidase (HRP) were used for specific protein bands detection with an ECL system. ImageJ software (the National Institutes of Health, USA) was used for protein band intensity analysis.

### 2.5. Quantitative Real-Time RT-PCR (qRT-PCR)

The RNeasy Mini Kit (#74106, Qiagen, Germantown, MD USA) was used for isolation of total RNA from cultured cells following the manufacturer’s instruction. The NanoDrop 2000c spectrophotometer (ND-2000c, Thermo Scientific, Wilmington, DE, USA) was employed to measure RNA concentrations. RNA samples were stored at −80 ℃. qRT-PCR was performed in a total volume of 20 µL using 10 µL of 2× Luna^®^ Universal One-Step Reaction Mix (#E3005, New England Biolabs, Ipswich, MA USA), 1 µL of RT Enzyme Mix (20×), 1 µL of 5 µM primer for each primer per reaction, 2 µL of the RNA dilution (100 ng/mL), and water. The following primers were used: (1) For the EGFR gene: Forward, 5′-CCA GTA TTG ATC GGG AGA GC-3′; reverse, 5′-CCA AGG ACC ACC TCA CAG TT-3′. (2) For the GAPDH gene: Forward, 5′-GGGAAGGTGAAGGTCGGAGT-3′; reverse, 5′-GGAGGGATCTCGCTCCTG-3′. The PCR cycling on a StepOnePlus^TM^ Real-Time PCR System (Applied Biosystems, Life Technologies, CA, USA) was performed as follows: A reverse transcription step (55 ℃, 15 min) and an initial denaturation step (95 ℃, 1min), followed by 45 cycles of denaturation (95 ℃, 10 s), extension (60 ℃, 60 s), and a single cycle of melting curve measurement step (95 ℃ for 15 s, 60 ℃ for 15 s, and 95 ℃ for 15 s). The fold-change for the expression level of EGFR mRNA relative to GAPDH mRNA was calculated using the 2^−∆∆Ct^ method, as previously described [24].

### 2.6. Immunohistochemical Analysis

Immunohistochemistry analysis was performed on 5 μm formalin-fixed paraffin-embedded tumor tissue sections. Anti-Ki-67 (#RB-9043-P0; Thermo Scientific, Fremont, CA, USA) and anti-cleaved poly (ADP ribose) polymerase 1 (cPARP) (#5625, Cell Signaling) were used as primary antibodies. Staining was performed by incubation for 5 min with diaminobenzidine (DAB) using a DAB peroxidase substrate kit (Vector Laboratories, Burlingame, CA, USA), as previously described [25].

### 2.7. In Vivo Xenograft Tumor Assays

All mouse experiments were conducted following Institutional Animal Care and Use Committee (IACUC) guidelines and approved protocols. NOD scid gamma mouse (NSG) mice (6- to 8-week-old) were used for xenograft studies. For subcutaneous xenografts, 5 × 10^6^ JHU-029 HNSCC cells suspended in 200 μL medium containing 45% Matrigel basement membrane matrix (#354234; BD Biosciences) were inoculated into the right flank of mice. Treatment with vehicle or hDT806 was started when the median tumor size reached approximately 80 mm^3^. hDT806 was administered via intratumoral injection at a dose of 12 μg/kg/mouse every other day. The tumor size was measured once every 2–3 days with a caliper. After 26 days of treatment, mice with tumors were euthanized and the tumors were dissected for analysis.

### 2.8. Statistical Analysis

All data are expressed as mean ± standard deviation. Student *t* test and one-way analysis of variance (ANOVA) were used where appropriate for statistical analysis. All tests were two-sided and *p* < 0.05 was considered significant.

## 3. Results

### 3.1. hDT806 Renders Potent Inhibition in Cell Viability and Proliferation of Human HNSCC Cell Lines

**Figure 1:**
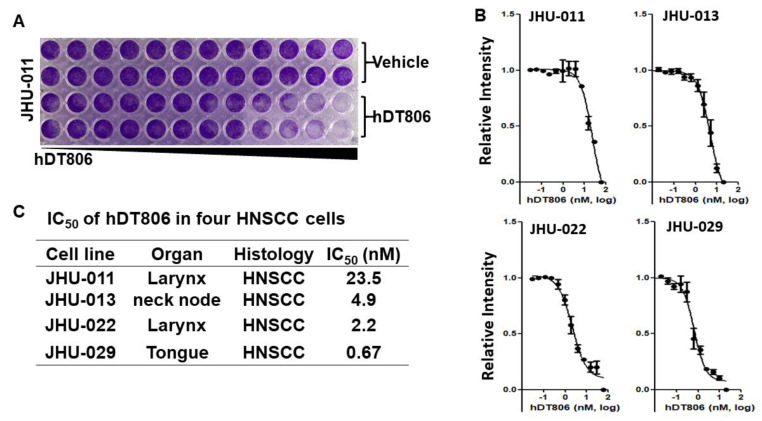
Sensitivity of HNSCC cells to hDT806. (**A**) Dose-escalation effects of hDT806 on cell viability in the JHU-011 HNSCC cells. The cells were exposed to vehicle or hDT806 with increased doses for 7 days. Data are shown of two duplicates. (**B**,**C**) IC50 of JHU-011, -013, -022, and -029 cells to hDT806. Data of four independent experiments are presented as mean ± SD (*n* = 4).

Previously, we generated a bivalent recombinant immunotoxin, hDT806, targeting EGFRvIII and overexpressed EGFR in cancers and demonstrated its high potency against glioblastoma cells with EGFR and EGFRvIII overexpression [15]. Since about 90% of HNSCC has EGFR overexpression [26], to test whether hDT806 exhibits efficacy against human HNSCC, we evaluated the effects of hDT806 on the viability and proliferation of four HNSCC cell lines: JHU-011, -013, -022, and -029 cells. All the four cell lines exhibited a suppressed viability and proliferation response. As shown in Figure 1A, hDT806 dose-dependently decreased the cell viability of the JHU-011 cells. In JHU-011, -013, -022, and -029, the dose-response cell viability and proliferation experiments in these cells revealed that hDT806 decreased cell viability with an IC50 value of 23.5, 4.9, 2.2, and 0.67 nM, respectively (Figure 1B,C).

The EGFR-specific tyrosine kinase inhibitor (TKI) erlotinib has been explored as an antitumor agent in HNSCC [27] and shows a selective efficacy in HNSCC patients [28,29]. To assess whether hDT806 shares a similar cytotoxicity to erlotinib, we carried out a parallel cell viability assay and measured the cytotoxicity of erlotinib in the same panel of HNSCC cells. The four cell lines demonstrated varied responses to erlotinib treatment, with an IC50 of 0.27 µM for JHU-011, 7.9 µM for JHU-013, 0.23 µM for JHU-022, and 43 µM for JHU-029, respectively (Supplementary Figure S1), suggesting that, while the HNSCC cells only respond to erlotinib at a micromolar scale, they are exquisitely sensitive to the EGFR-targeting hDT806 at a nanomolar scale. We also tested the four cell lines’ responses to cetuximab, the only FDA-approved anti-EGFR targeted therapy in the clinic. Cetuximab treatment rendered an IC50 of 35.2 µg/mL for JHU-011, 50.5 µg/mL for JHU-013, 101.4 µg/mL for JHU-022, and 103.3 µg/mL for JHU-029, respectively (Supplementary Figure S2), indicating that JHU-029 and -022 are not very sensitive to cetuximab in the four cell lines. Our data support the notion that hDT806 has an anticancer activity that is distinct from erlotinib or cetuximab. For the JHU-029 cells that were not sensitive to either erlotinib or cetuximab were highly sensitive to hDT806. Therefore, we employed JHU-029 to study the mechanism of hDT806-induced cytotoxicity.

### 3.2. hDT806 Decreases EGFR Protein Levels and Disrupts Its Downstream Effectors in HNSCC Cells

**Figure 2:**
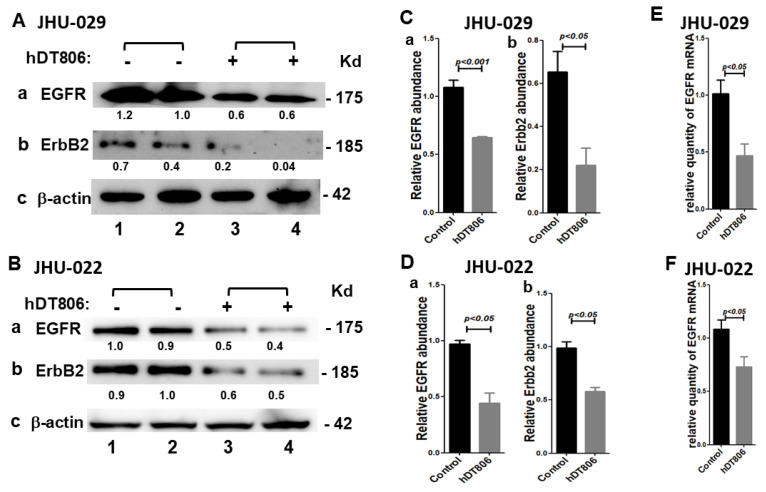
hDT806 decreases EGFR and ErbB2 in JHU-029 and JHU-022 cells. (**A**–**D**) Total protein extracts were prepared from the cells treated with vehicle or hDT806 (20 nM). Western blot analysis was performed for EGFR (**Aa**), ErbB2 (**Ab**), and β-actin (**Ac**) in JHU-029 (*n* = 4) and EGFR (**Ba**), ErbB2 (**Bb**), and β-actin (**Bc**) in JHU-022 (*n* = 3) cells treated with vehicle or hDT806 for 48 h, respectively. (**C**,**D**) Protein band intensities relative to the corresponding β-actin bands were quantified in JHU-029 (**C**) and JHU-022 (**D**) for comparisons between the vehicle-treated cells and the hDT806-treated cells. (**E**,**F**) Total RNA extracts were prepared from the cells treated with vehicle or hDT806. Real-time reverse transcription quantitative PCR analysis was performed to evaluate EGFR mRNA expression in JHU-029 (**E**) and JHU-022 (**F**) cells treated with vehicle or hDT806 for 48 h (*n* = 4), respectively. Data of three or four independent experiments are presented as mean ± SD.

hDT806 was generated to capitalize the specificity of mAb806 to the overexpressed EGFR and EGFRvIII variant [15]. It iss known that surface receptors can provide an efficient gateway for the internalization of anti-receptor targeting immunotoxins and downregulate the receptors [30]. To explore the mechanism of the inhibition of cell proliferation by hDT806, we first assessed EGFR expression levels. Indeed, we found that 48 h treatment of hDT806 significantly reduced EGFR protein levels by 39.5 ± 4.4% in the JHU-029 cells (Figure 2(Aa,Ca); Figure S3A), and 54.6 ± 4.4% in the JHU-022 cells (Figure 2(Ba,Da); Figure S3B). Consistent with the downregulation of EGFR protein levels, EGFR gene expression was also affected and decreased by 53.6 ± 0.9% in JHU-029 (Figure 2E) and 32.2 ± 0.9% in JHU-022 (Figure 2F) after treatment with hDT806, respectively. Interestingly, another HER/ErbB family surface receptor, ErbB2, was also affected by hDT806 treatment in HNSCC, showing a significant decrease in ErbB2 protein levels, by 65.8 ± 9.9% (Figure 2(Ab,Cb); Figure S3A) in JHU-029 and 40.2 ± 7.3% (Figure 2(Bb,Db); Figure S3B) in JHU-022, respectively.

**Figure 3:**
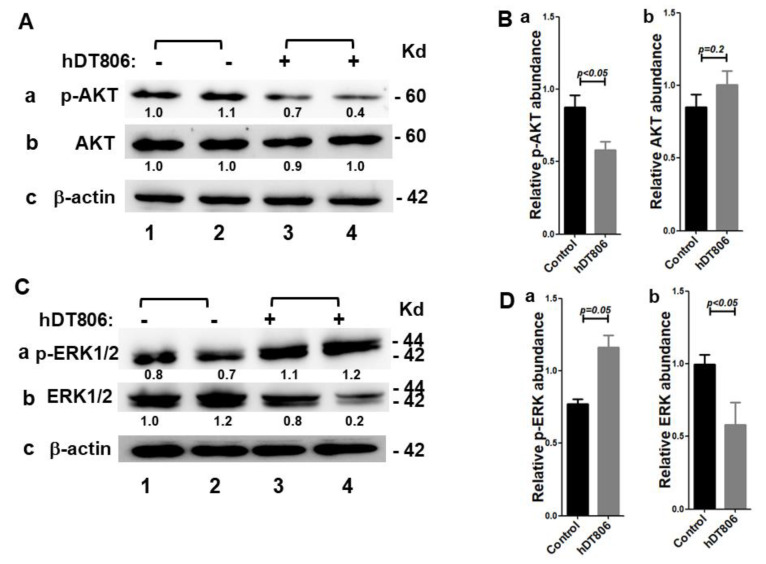
hDT806 disrupts EGFR signaling and the downstream effectors, ERK1/2 as well as AKT in JHU-029 cells. (**A**) Total protein extracts were prepared from the cells treated with vehicle or hDT806 (20 nM). Western blot analysis was performed for *p*-AKT (**a**), AKT (**b**), and β-actin (**c**) in the cells treated with vehicle or hDT806 for 48 h. (**B**) Protein band intensities relative to the corresponding β-actin bands were quantified for comparisons between the vehicle-treated cells and the hDT806-treated cells. Data of four independent experiments are presented as mean ± SD (*n* = 4). (**C**) Total protein extracts were prepared from the cells treated with vehicle or hDT806 (20 nM). Western blot analysis was performed for *p*-ERK1/2 (**a**), ERK1/2 (**b**), and β-actin (**c**) in the cells treated with vehicle or hDT806 for 48 h. (**D**) Protein band intensities relative to the corresponding β-actin bands were quantified for comparisons. Data of four independent experiments are presented as mean ± SD (*n* = 4).

Next, we analyzed the effects of hDT806 on EGFR downstream effectors in the JHU-029 cells. The EGFR is activated by ligands including EGF and transforming growth factor (TGFα and β), resulting in the auto-phosphorylation of the intracellular domain with downstream activation of PI3K/AKT and mitogen activated protein kinase (MAPK) pathways to elicit survival and proliferation. Western blot analysis revealed that both the classic EGFR downstream effector AKT protein and extracellular signal-regulated kinase1/2 (ERK1/2) protein of the MAPK pathway were disrupted by the treatment of hDT806. As shown in Figure 3, we found a great decrease of 31.2 ± 9.5% in the level of phospho-AKT by hDT806 (Figure 3(Aa,Ba); Figure S4A); however, the level of AKT protein was not altered significantly (Figure 3(Ab,Bb); Figure S4A). To our surprise, ERK1/2 showed a distinct response to the treatment of hDT806. The level of phospho-ERK1/2 increased by 50.8 ± 17.3% in the hDT806-treated cells compared with that of the vehicle-treated JHU-029 (Figure 3(Ca,Da); Figure S4B), while the level of ERK1/2 protein was significantly suppressed by 40.8 ± 15.6% in hDT806-treated cells compared to vehicle-treated cells (Figure 3(Cb,Db); Figure S4B).

These results indicate that by targeting the EGFR on the HNSCC cells, hDT806 disrupts EGFR signaling and its downstream effectors, which may lead to inhibition in cell proliferation in HNSCC cells.

### 3.3. hDT806 Affects Transcription by Inhibiting RNA Polymerase II Phosphorylation in HNSCC Cells

**Figure 4:**
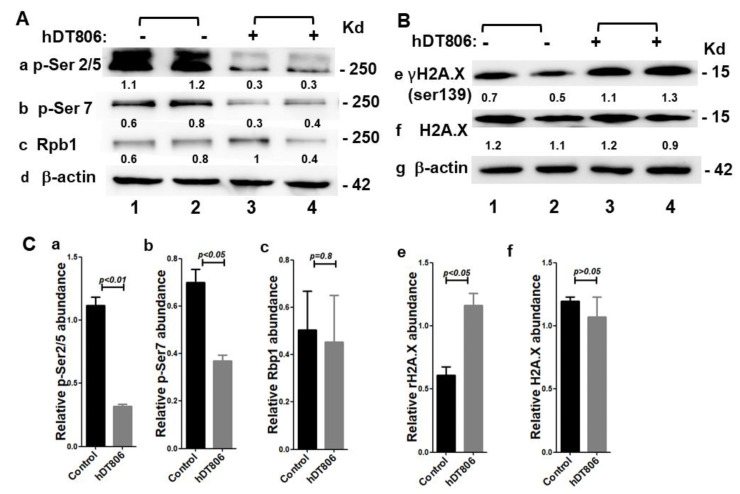
hDT806 induces transcription inhibition and DNA damage. (**A**) Total protein extracts were prepared from the cells treated with vehicle or hDT806 (20 nM). Western blot analysis was performed for RNAPII CTD *p*-Ser2/5 (**a**), RNAPII CTD *p*-Ser7 (**b**), RNAPII large subunit Rpb1 (**c**), and β-actin (**d**) in the cells treated with vehicle or hDT806 for 48 h. (**B**) Western blot analysis was performed for γH2A.X (**e**), H2A.X (**f**), and β-actin (**g**). (**C**) Band intensities of *p*-Ser2/5 (**a**), *p*-Ser7 (**b**), RNAPII large subunit Rpb1 (**c**), γH2A.X (**e**), and H2AX (**f**) were quantified relative to the corresponding β-actin bands for comparisons. Data of three independent experiments are presented as mean ± SD (*n* = 4).

Since hDT806 reduced the EGFR mRNA transcript, we evaluated the effect of hDT806 on gene transcription. DT is known to kill cells by ADP-ribosylation on the unique diphthamide residue of the elongation factor eEF2, leading to a defect in translation elongation and protein synthesis inhibition [31]. However, it remains less certain whether immunotoxin directly affects cellular transcription activity. During gene expression, the carboxyl-terminal domain (CTD) of the RNA polymerase II large subunit Rpb1 undergoes sequential phosphorylation on different residues by a set of CDKs [32,33]. To test whether hDT806 may cause disruption to protein synthesis by the affecting cellular transcription process, we examined the effects of hDT806 on the CTD phosphorylation of Rpb1 in JHU-029 after the cells were treated with vehicle or hDT806 for 48 h. After hDT806 treatment, the levels of phosphorylation at the site of Rpb1 CTD Ser2/5 and Ser7 were reduced to 28.7 ± 3.5% (Figure 4(Aa,Ca); Figure S5A) and 53.4 ± 7.6% (Figure 4(Ab,Cb); Figure S5A) of that with vehicle treatment, respectively, while the level of Rpb1 remained unchanged in the JHU-029 cells (Figure 4(Ac,Cc); Figure S5A). These data indicate that hDT806 effectively inhibits RNA polymerase II CTD phosphorylation to inhibit the transcription process without affecting the RNA polymerase II large subunit Rpb1 in the HNSCC JHU-029 cells.

### 3.4. hDT806 Induces DNA Damage Responses in HNSCC Cells

The DNA damage response can be initiated following a variety of stress signals, such as DNA-damaging therapeutic agents. The EGFR is known to steer the pathways related to proliferation, DNA damage repair, and apoptosis in HNSCC [34]. The targeting of the EGFR signaling pathway was found to decrease the repair capacity of DNA double-strand break (DSB), the most deleterious type of DNA damage, in tumor cells [35]. It is known that the phosphorylation of histone H2A variant H2A.X at Ser 139 (γH2A.X) is well correlated with DSB and considered as the most sensitive marker for DNA damage [36]. We next tested whether EGFR-targeted hDT806 induces a DNA damage response by evaluating the γH2A.X level. After hDT806 treatment for 48 h, we found that the level of γH2A.X was increased to 194.7 ± 37% (Figure 4(Be,Ce); Figure S5B) of that with vehicle treatment. However, hDT806 treatment did not change the level of H2A.X (Figure 4(Bf,Cf); Figure S5B). Thus, the data indicate that by targeting overexpressed EGFR, hDT806 treatment causes DNA damage in the HNSCC cells.

### 3.5. hDT806 Activates Apoptosis Pathways and Induces Apoptosis in HNSCC Cells

**Figure 5:**
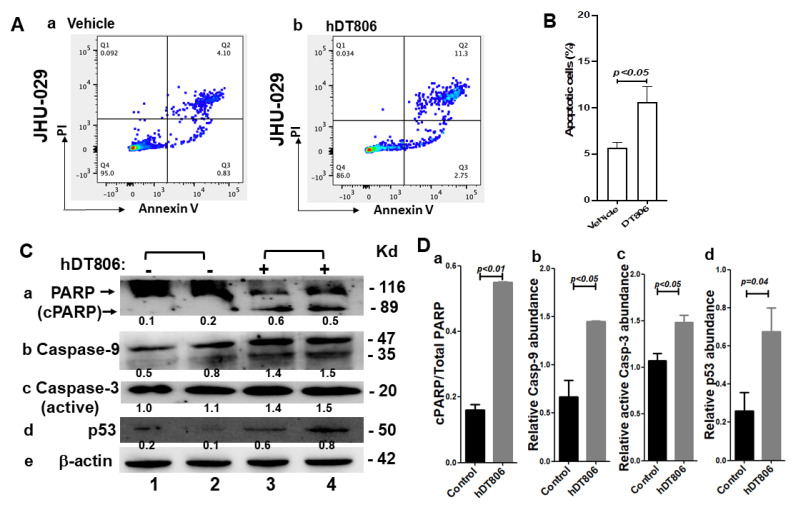
hDT806 treatment induces apoptosis in the JHU-029 cells. (**A**) Cells were treated with vehicle (**a**) or 20 nM hDT806 (**b**) for 48 h and collected for annexin V and PI staining followed by flow cytometric analysis for apoptotic cells in JHU-029. (**B**) The populations of apoptotic cells were quantified for JHU-029. Data of four independent experiments are presented as mean ± SD (*n* = 4). (**C**) Total protein extracts were prepared from the cells treated with vehicle or hDT806 (20 nM) for 48 h. Western blot analysis was performed for PARP and cleaved PARP (**a**), p53 (**b**), caspase-9 (**c**), active caspase-3 (**d**), and β-actin (**e**). (**D**) Relative band intensities of cleaved PARP/PARP (**a**), band intensities of p53 (**b**), caspase-9 (**c**), and active caspase-3 (**d**) were quantified relative to the corresponding β-actin bands for comparisons. Data of three independent experiments are presented as mean ± SD (*n* = 3).

Many anticancer drugs exert their cytotoxicity through DNA damage and apoptosis induction [37]. Apoptosis is one of the mechanisms underlying cell proliferation control. We proceeded to evaluate apoptotic events in the JHU-029 cells by flow cytometry analysis. With the treatment of hDT806 for 48 h, we found that the apoptotic cells were significantly increased from 5.8% with vehicle treatment (Figure 5(Aa,B)) to 10.7% with hDT806 treatment (Figure 5(Ab,B)). Thus, the cytotoxicity of hDT806 may involve the ability of hDT806 to induce cell apoptosis.

It is well known that apoptosis can be triggered by the extrinsic (the death receptor) pathway and the intrinsic (the mitochondrial) pathway, with both converging upon the activation of the caspase protease family, leading to the dismantling of the cell [38]. To investigate the pathways involved the apoptotic events detected in flow cytometric analysis, we performed western blot analysis to determine which apoptotic proteins were changed upon the treatment (Figure 5C,D; Figure S6A–C). In JHU-029, the levels of the proapoptotic proteins we investigated were increased by hDT806 treatment, with the ratio of cleaved PARP relative to PARP (cPARP/PARP) increased to 6.4 ± 0.9 folds (Figure 5(Ca,Da)), the level of caspase-9 increased to 2.3 ± 0.6 folds (Figure 5(Cb,Db)), and the level of active caspase-3 increased to 1.4 ± 0.1 folds (Figure 5(Cc,Dc)), of that in vehicle-treated cells, respectively. The levels of p53 protein in unstressed cells are very low because it is targeted for proteasomal degradation, and the *TP53* gene is activated in response to many stress stimuli such as oncogene activation and DNA damage [39]. Indeed, in our experiment, the treatment of hDT806 increased the pro-apoptotic protein p53 to 3.7 ± 2.6 folds (Figure 5(Cd,Dd)) of that of vehicle treatment, with the latter being low.

Consistent with the flow cytometry analysis, these results indicated that hDT806 treatment results in increased apoptotic proteins and triggers apoptotic cell death in HNSCC cells.

### 3.6. In Vivo hDT806 Administration Inhibits the Growth of JHU-029 Tumors in a Mouse Model Involving Apoptosis Induction and Growth Inhibition

**Figure 6:**
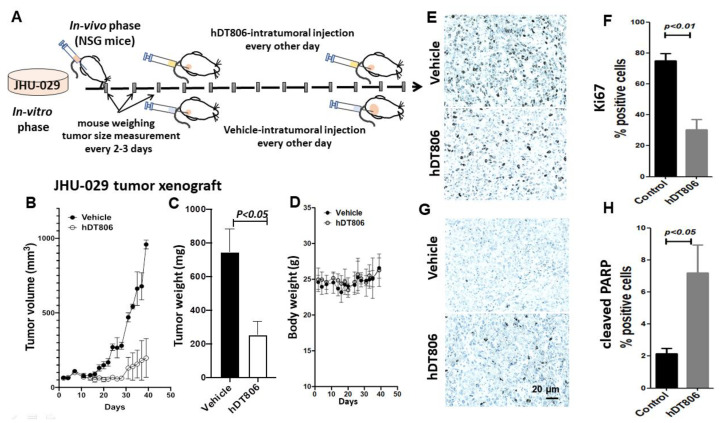
Intratumoral injection of hDT806 treatment inhibits tumor growth of JHU–029 cells in NSG mice. (**A**) A schematic diagram shows the treatment procedure. When the tumors reached an average size of ~80 mm3, intratumoral injection of vehicle or hDT806 was administered for 26 days. (**B**) Tumor growth curves plotted for the treatment with vehicle or hDT806. (**C**) Average weight of the dissected tumors from NSG mice treated with vehicle or hDT806 after 26-day treatment. (**D**) Average body weight of the mice treated with vehicle or hDT806 during the treatment course. hDT806 treatment reduces Ki67 (**E**,**F**) and increases cleaved PARP (**G**,**H**) in the JHU-029 xenograft tumors. Scale bar = 20 µM. Data are presented as mean ± SEM (*n* = 4).

To investigate whether in vivo hDT806 treatment could recapitulate its anti-HNSCC efficacy in in vitro settings, we proceeded to assess the effects of hDT806 treatment in an HNSCC xenograft tumor model of JHU-029 in NSG mice (Figure 6A). hDT806 was administered via intratumoral injection. As shown in Figure 6B, the tumor volume of the mice treated with vehicle continuously increased, while the tumor volumes of the mice treated with hDT806 were stabilized 6 days after treatment, although the tumors exhibited increased volumes at the late stage. Compared with the vehicle-treated mice, the hDT806-treated mice showed a significant suppression of the tumor growth (Figure 6B) as well as the tumor weight (Figure 6C). We did not observe a significant difference in the body weight between the vehicle-treated and hDT806-treated mice during the course of treatment (Figure 6D). The in vivo data support the notion that hDT806 effectively inhibits the growth of HNSCC tumors in mice.

To explore the mechanisms of the hDT806-induced inhibition of tumor growth, we examined cancer cell proliferation in the JHU-029 xenograft tumors. The effectiveness of the treatment was validated by immunohistochemical analysis of the xenograft tumor tissues. We measured crucial proteins for cell proliferation and apoptosis, Ki67 and cleaved PARP, respectively. Without hDT806, numerous Ki67-postive cells were found in the JHU-029 tumor tissues. After hDT806 treatment, the Ki67-postive cells were significantly reduced from 75 ± 4.7% in vehicle-treated to 30.3 ± 6.6% in hDT806-treated tumors (Figure 6D,E). On the contrary, the number of cleaved PARP-positive cells was significantly increased from 2.1 ± 0.3% in vehicle-treated to 7.2 ± 1.7% in hDT806-treated tumors (Figure 6F,G). Thus, in line with our in vitro experiments, the in vivo results support the notion that hDT806 inhibits tumor growth of the JHU-029 cells involving apoptosis induction.

## 4. Discussion

Here, we demonstrated the remarkable antitumor activities of the recombinant immunotoxin hDT806 to suppress HNSCC in both in vitro and in vivo settings. In four HNSCC lines (JHU-011, -013, -022, and -029), EGFR-targeted hDT806 effectively suppressed the growth and proliferation of HNSCC cells with the IC50s ranging between 0.7–24 nM. In an HNSCC xenograft model of JUH-029 cells that are insensitive to erlotinib or cetuximab, the intra-tumor injection of hDT806 substantially reduced the tumor mass. These data support the notion that EGFR-targeted hDT806 may exhibit a significant tumor-suppression efficacy in HNSCC.

The development of recombinant immunotoxin therapy has been driven by its unique features: its high specificity, extraordinary potency, effectiveness against quiescent non-dividing cells, and the lack on any known cross-resistance with other agents [40]. DT is known to be extreme toxic, to the degree that even a single DT molecule is enough to kill one cell. Various types of cancer are known to have oncogenic EGFR alterations, including EGFR overexpression, gene amplification, and tumor-specific mutation. For example, glioblastoma harbors diverse EGFR genetic alterations, with the mutant EGFRvIII as a tumor-specific surface marker [41,42]. Recognizing the unique specificity of mAb806 to overexpressed EGFR and EGFRvIII mutant [18,19], to steer the potent cytotoxicity of DT specifically to cancer cells while sparing normal cells, we previously generated hDT806 by fusing an engineered DT fragment, DT390, with two single chain variable fragments of mAb806 targeting overexpressed EGFR and/or EGFRvIII, and demonstrated the efficacy of hDT806 in glioblastoma, especially those with EGFRvIII expression [15]. Of HNSCC, 90% contains overexpressed EGFR [26]. Indeed, our current in vitro and in vivo experiments showed a potent cytotoxicity of hDT806 in HNSCC cells. Among the earliest targeted therapies that block growth signals, TKIs and monoclonal antibodies are two main classes of EGFR inhibitors used in clinical settings. However, over the years, both have manifested various primary and/or acquired therapy resistance mechanisms in different solid tumors, dampening their efficacy. Erlotinib, the first FDA-approved EGFR-TKI for treating *EGFR*-mutated non-small cell lung cancer and pancreatic cancer, shows efficacy in some HNSCC patients [28,43]. Cetuximab is the only FDA-approved anti-EGFR targeted therapy in HNSCC. In this study, we showed one particularly interesting result of JHU-029; although the cells showed a primary resistance to erlotinib (IC50 = 43 µM) and insensitivity to cetuximab (IC50 = 103.3 µg/mL) among the four HNSCC cells we tested, JHU-029 was exquisitely sensitive to hDT806 treatment (IC50 = 0.67 nM). Our data provide a direct line of evidence to support the potential of the immunotoxin hDT806 as an effective alternative therapeutic agent in treating HNSCC.

In the current research, we demonstrated several integrative aspects of the cytotoxicity of the bivalent, bispecific hDT806 targeting overexpressed and/or EGFRvIII to HNSCC cells. Western blot analysis showed that hDT806 reduced EGFR protein levels and disrupted the downstream effectors of EGFR signaling, including MAPK/ERK1/2 and AKT proteins. The EGFR is known to regulate cellular activities including cell cycle progression, proliferation, anti-apoptosis, and migration. The EGFR was found to regulate DNA damage repair mediated via PI3K/AKT and ERK1/2 pathways in cancer [44]. In HNSCC cells, hDT806-induced apoptotic cell death was revealed using flow cytometric analysis. We further found that hDT806 treatment increased apoptotic proteins, such as the initiator caspase caspase-9, the executioner caspase caspase-3, p53, as well as the cleaved PAPR. It is well known that RITs based on DT or *Pseudomonoas* exotoxin (PE) inhibit protein synthesis by ADP-ribosylation [30], and our research further showed that hDT806-triggered a drastic inhibition in RNA polymerase II carboxy-terminal domain phosphorylation and a significantly increase in γH2A.X levels, indicating a direct transcription inhibition and DNA damage imposed on HNSCC cells by hDT806. The in vitro growth inhibition and apoptosis induction by hDT806 in HNSCC were recapitulated in a mouse JUH-029 xenograft model, in which treatment with hDT806 was shown to reduce Ki-67 and increase cleaved PARP proteins using IHC analysis. Cancer is known for its hallmark biological alterations acquired during the multistep tumorigenesis, including sustaining proliferative signaling, evading growth suppressors, and resisting cell death, among others [45]. Together, our data indicate that EGFR-targeted hDT806 inhibits cancer cell proliferation and suppresses the growth of xenograft tumors by interfering with multiple cellular processes, such as the disruption of EGFR signaling, inhibition of transcription, DNA damage, as well as apoptotic response.

We obtained the in vivo antitumor effects of hDT806 via intratumoral injection in the mouse HNSCC model. This drug administration strategy may circumvent a potential issue of the neutralizing antibody to RIT if hDT806 is administered in immunocompetent mice. It is reported that after treatment is initiated, rapidly developed anti-toxin antibodies bind to RIT to prevent it from killing tumor cells [40]. Since RIT produces complete regression and prolongs survival, it has been approved by the FDA to treat several types of hematopoietic malignancies [8,9,10,11,12,46]. However, RIT has only limited applicability for solid tumors due to its inherent immunogenicity and toxicity. Several approaches have been adopted to reduce immunogenicity, including approaches to prevent B cell activation by eliminating B cell epitopes [47], to prevent helper T cell activation by interfering with major histocompatibility complex II presentation or T cell recognition [48,49,50], or to suppress the host immune system [51]. While these approaches are effective, they fail to completely eliminate the immunogenicity of RIT. The delivery of a gene encoding RIT under a tumor-specific promoter has also been exploited to directly produce RIT in vivo to avoid immunogenicity [52,53]. The main challenges associated with gene-based RIT therapeutics are the efficient delivery and specific expression of RIT in all of the tumor tissues. Thus, to exploit the potent cytotoxicity of DT-based RIT, the development more effective delivery approaches in future research is needed for the prospective therapeutic usage of RIT in the treatment of solid tumors, including HNSCC.

## 5. Conclusions

EGFR-targeted recombinant immunotoxin hDT806 exhibits significant antitumor activities in HNSCC, causing the direct disruption of EGFR signaling; hDT806 further induces transcription inhibition, DNA damage, as well as apoptotic responses, which, in turn, may contribute to the antitumor efficacy of hDT806 distinct from erlotinib or cetuximab.

## Data Availability

The data presented in this study are included in this published article or in the Supplementary Materials.

## Supplementary Materials

The following are available online at https://www.mdpi.com/article/10.3390/biology11040486/s1, Figure S1: Sensitivity of HNSCC cells to cetuximab (µg/mL); Figure S2: Sensitivity of HNSCC cells to cetuximab (µg/mL); Figure S3: Analysis of hDT806 decreasing EGFR and ErbB2 in JHU-029 and JHU-022 cells; Figure S4: Analysis of hDT806 disruption of EGFR signaling and the downstream effectors, AKT as well as ERK1/2 in JHU-029 cells; Figure S5: Analysis of hDT806 inducing transcription inhibition and DNA damage; Figure S6: Ananlysis of apoptosis induced by hDT806 treatment in the JHU-029 cells.
