# Supplementary material for: Antitumor Efficacy of EGFR-Targeted Recombinant Immunotoxin in Human Head and Neck Squamous Cell Carcinoma
Source: Biology (Basel). 2022 Mar 22;11(4):486. doi: 10.3390/biology11040486 (PMC9027470; doi:10.3390/biology11040486)
Supplement: Supplementary file 1 [file biology-11-00486-s001.zip › biology-1625228-supplementary Figures S1 and S2.pdf]

Supplementary Figure S1

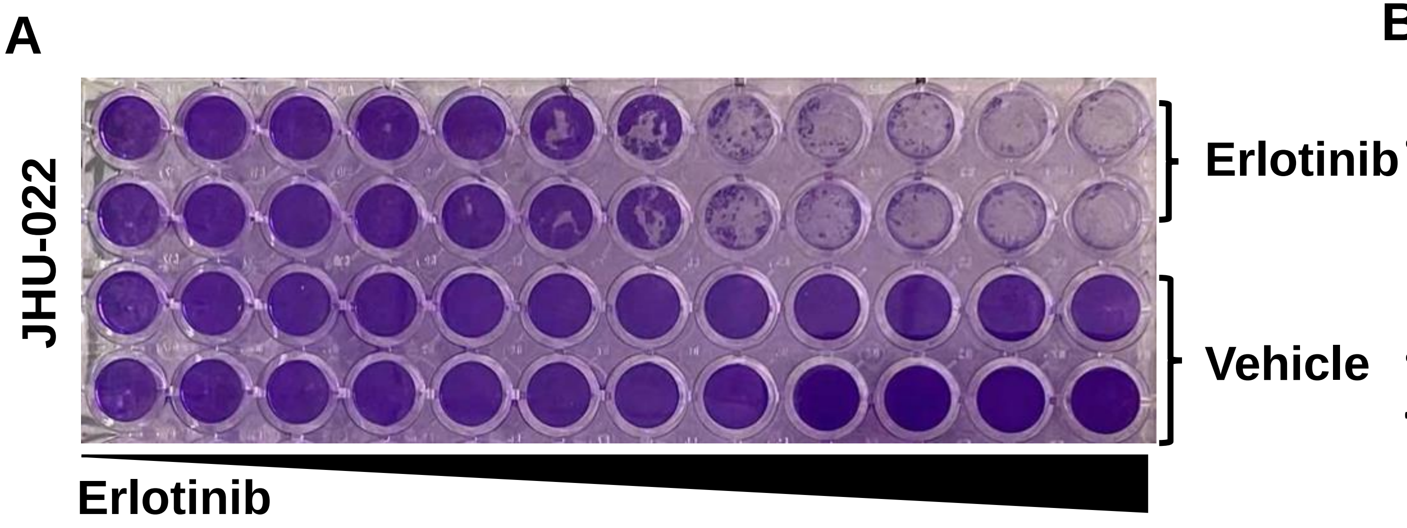

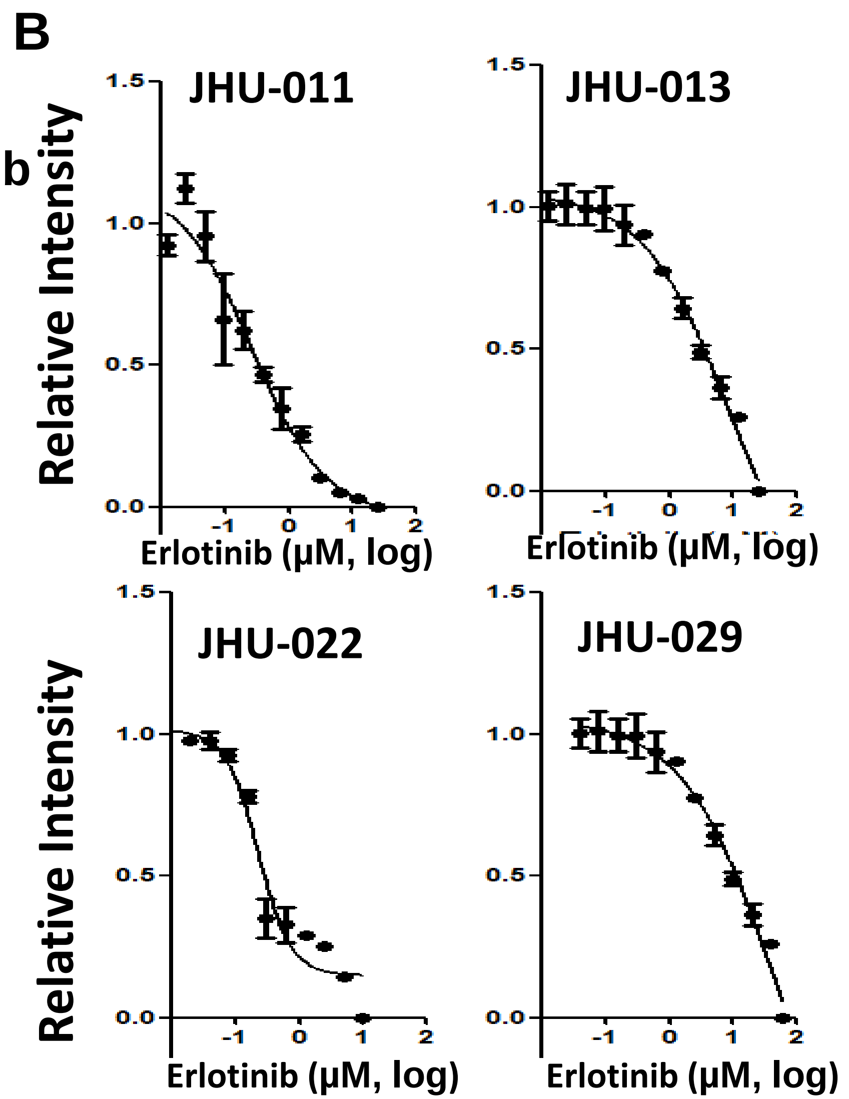

C **IC50 of erlotinib in four HNSCC cells**

Cell line	Organ	Histology	IC50 (μM)
JHU-011	Larynx	HNSCC	0.27
JHU-013	neck node	HNSCC	7.9
JHU-022	Larynx	HNSCC	0.23
JHU-029	Tongue	HNSCC	43

Figure S1 Sensitivity of HNSCC cells to erlotinib (μM). (A) Dose-escalation effects of erlotinib on cell viability in the JHU-022 HNSCC cells. The cells were exposed to vehicle or erlotinib with increased doses for 7 days. Data are shown of two duplicates. (B, C) IC50 of JHU-011,
-013, -022 and -029 cells to erlotinib. Data of four independent experiments are presented as mean ± SEM (n= 4).

Supplementary Figure S2

A

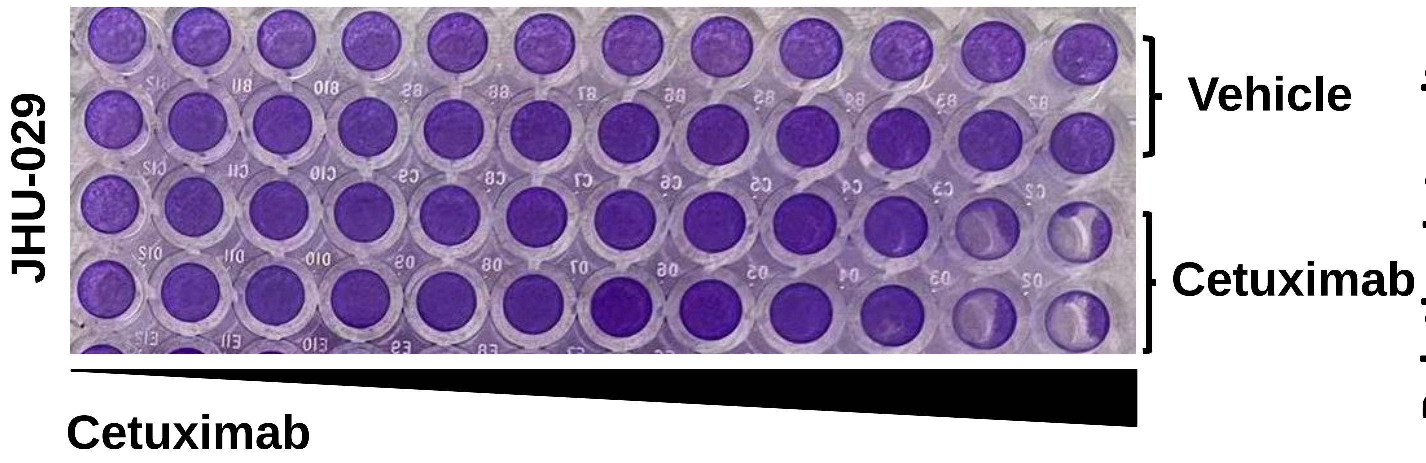

B

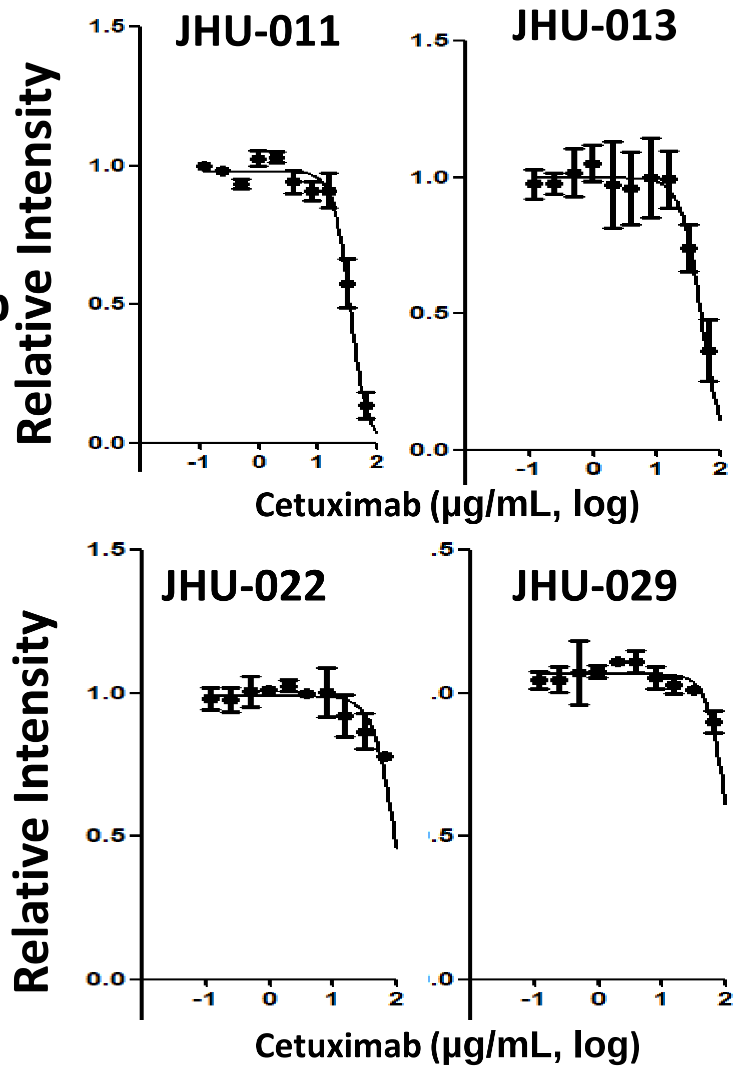

C

IC50 of cetuximab in four HNSCC cells

Cell line	Organ	Histology	IC50 (μg/mL)
JHU-011	Larynx	HNSCC	35.2
JHU-013	neck node	HNSCC	50.5
JHU-022	Larynx	HNSCC	101.4
JHU-029	Tongue	HNSCC	103.3

Figure S2 Sensitivity of HNSCC cells to cetuximab (μg/mL). (A) Dose-escalation effects of cetuximab on cell viability in the JHU-029 HNSCC cells. The cells were exposed to vehicle or cetuximab with increased doses for 7 days. Data are shown of two duplicates. (B, C) IC50 of JHU-011, -013, -022 and -029 cells to cetuximab. Data of four independent experiments are presented as mean ± SEM (n= 4).
