# Supplementary material for: Antitumor Efficacy of EGFR-Targeted Recombinant Immunotoxin in Human Head and Neck Squamous Cell Carcinoma
Source: Biology (Basel). 2022 Mar 22;11(4):486. doi: 10.3390/biology11040486 (PMC9027470; doi:10.3390/biology11040486)
Supplement: Supplementary file 1 [file biology-11-00486-s001.zip › biology-1625228-supplementary Figures S3-S6.pdf]

Supplementary Materials:

A

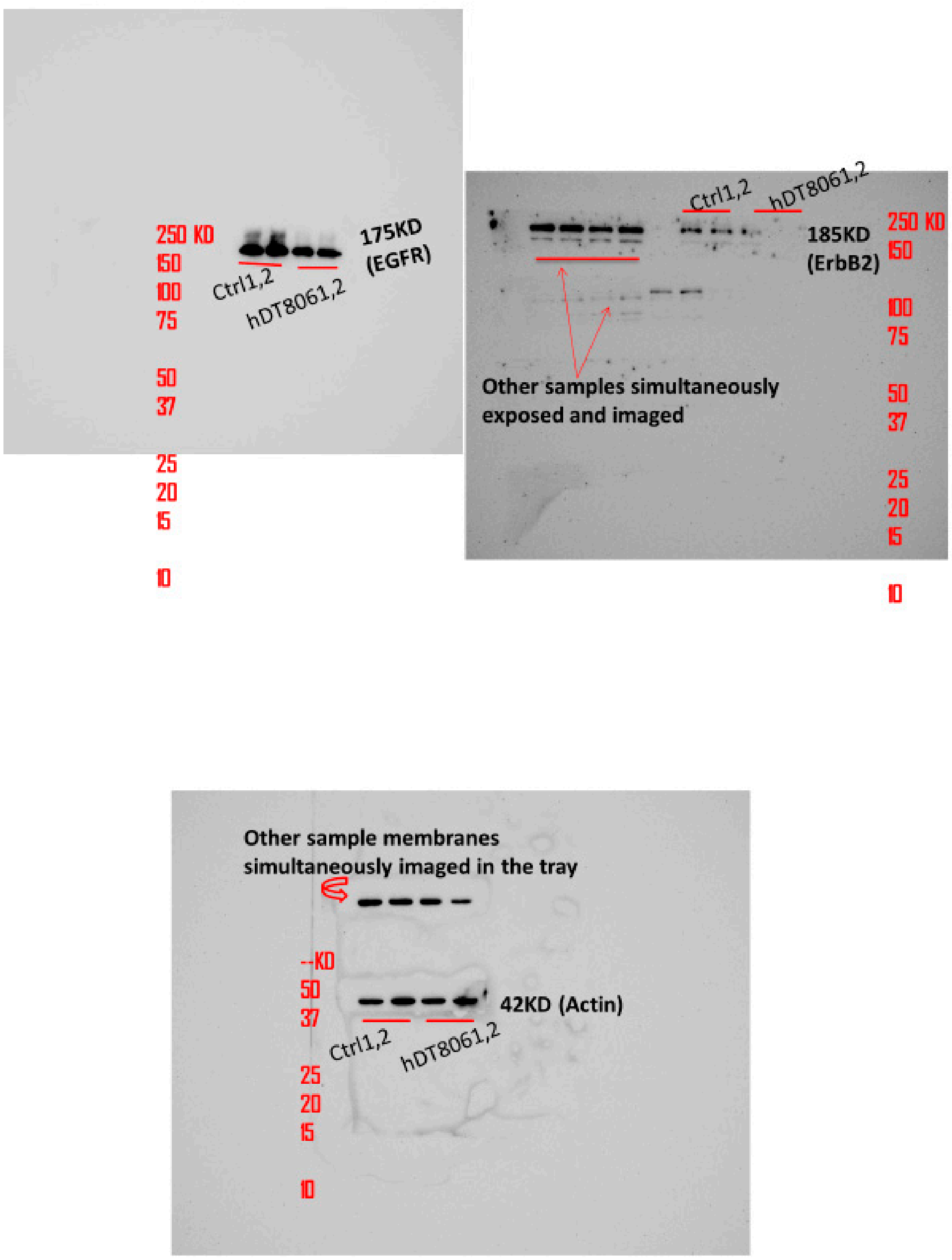

B

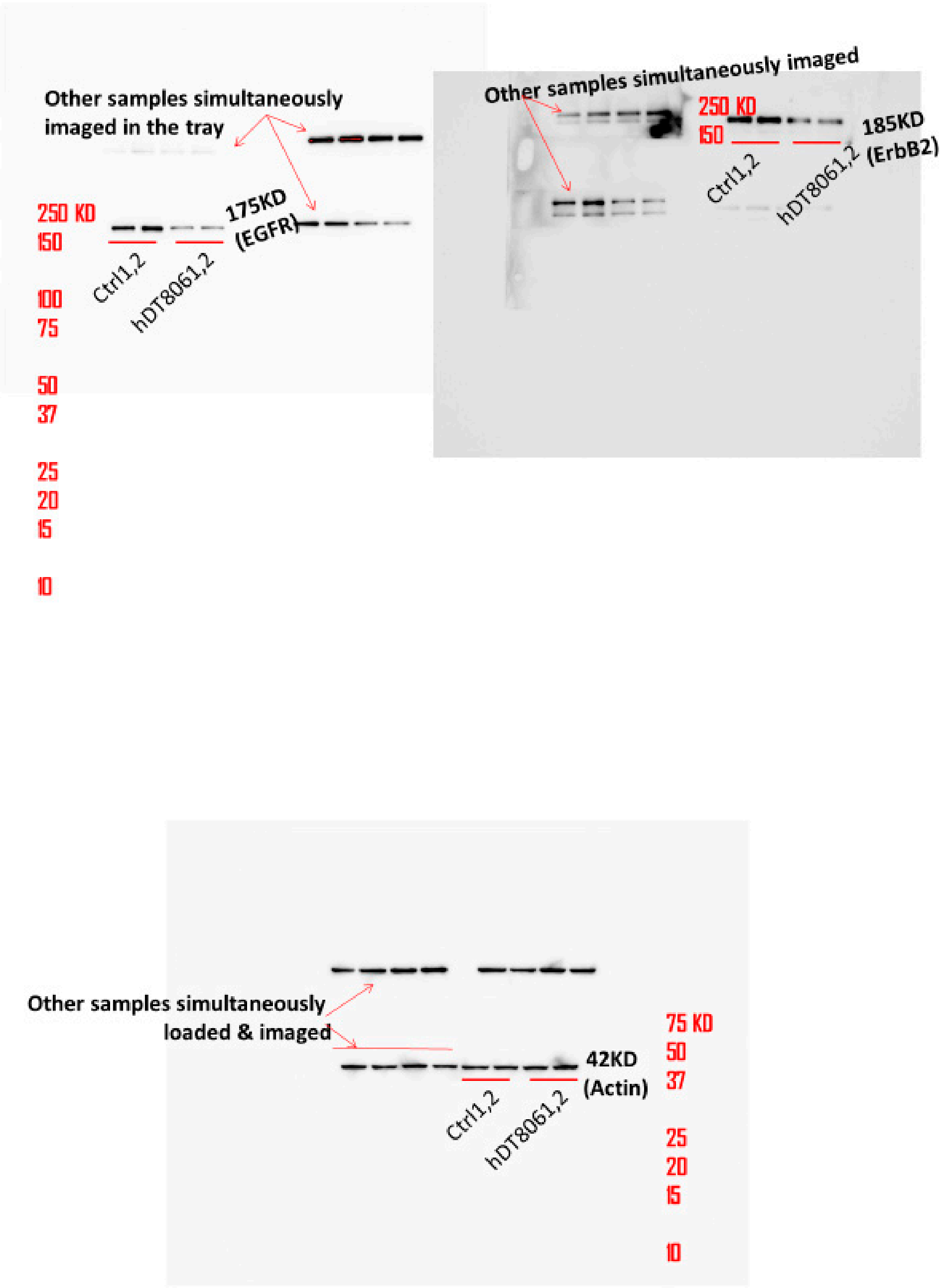

Figure S3: Analysis of hDT806 decreasing EGFR and ErbB2 in JHU-029 and JHU-022 cells.

A

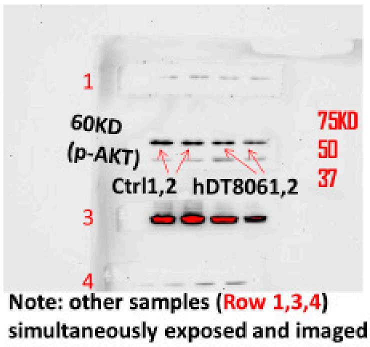
1
60KD
(p-AKT)
75KD
50
37
Ctrl1,2
hDT8061,2
3
4
Note: other samples (Row 1,3,4)
simultaneously exposed and imaged

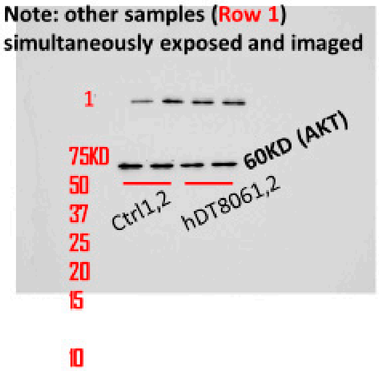
Note: other samples (Row 1)
simultaneously exposed and imaged
1
75KD
50
37
25
20
15
10
60KD (AKT)
Ctrl1,2
hDT8061,2

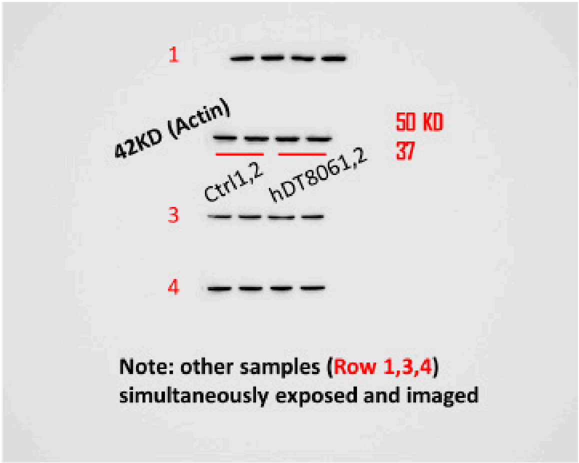
1
42KD (Actin)
50 KD
37
Ctrl1,2
hDT8061,2
3
4
Note: other samples (Row 1,3,4)
simultaneously exposed and imaged

B

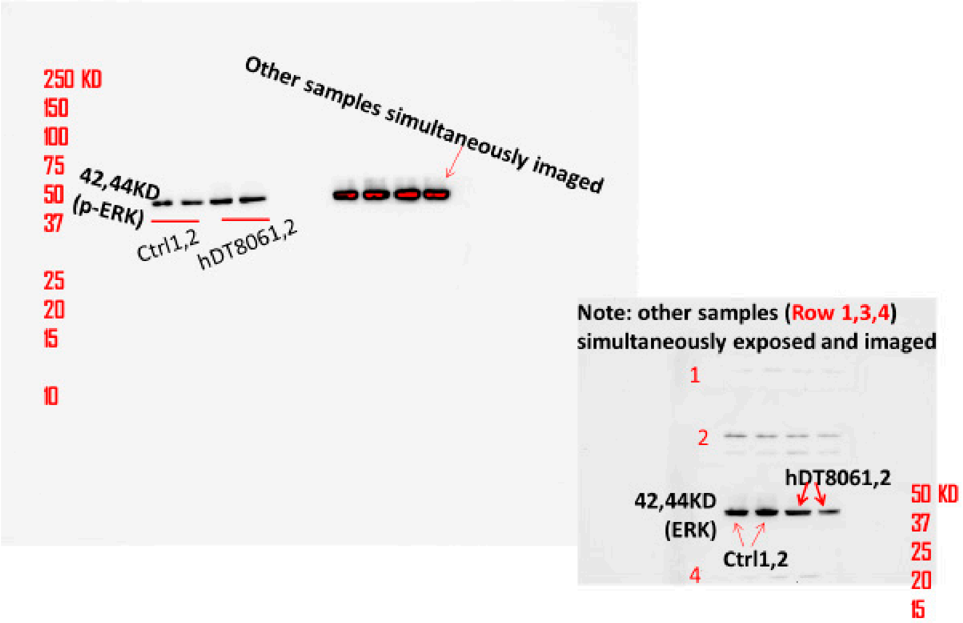

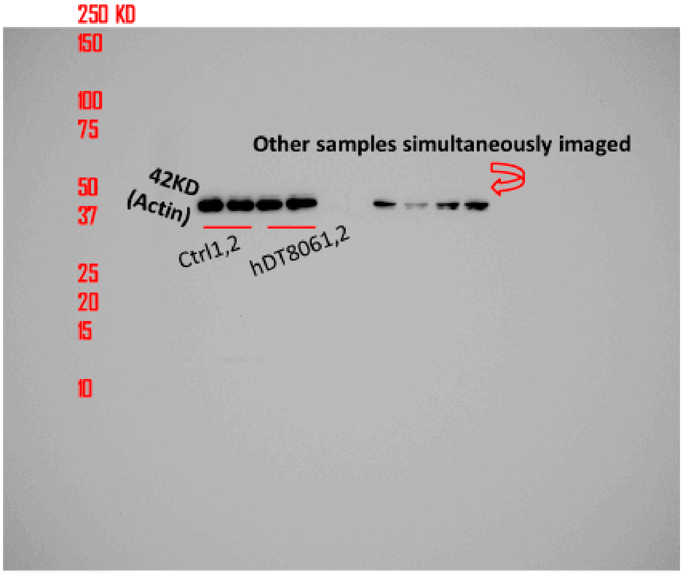

Figure S4: Analysis of hDT806 disruption of EGFR signaling and the downstream effectors, AKT as well as ERK1/2 in JHU-029 cells.

A

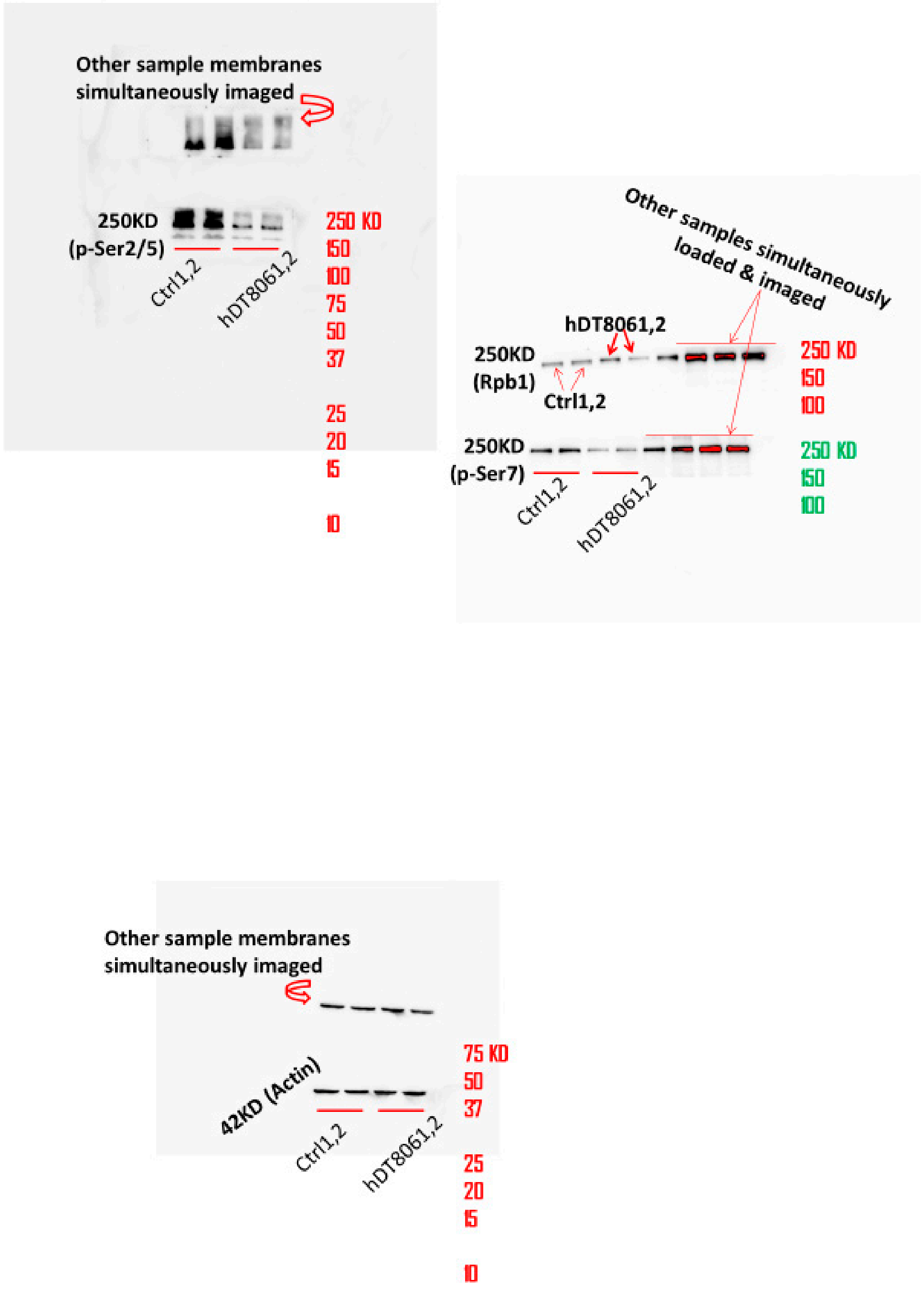
Other sample membranes simultaneously imaged
250KD (p-Ser2/5)
Ctrl1,2
hDT8061,2
250 KD
150
100
75
50
37
25
20
15
10
Other samples simultaneously loaded & imaged
hDT8061,2
250KD (Rpb1)
Ctrl1,2
250 KD
150
100
250KD (p-Ser7)
Ctrl1,2
hDT8061,2
250 KD
150
100
Other sample membranes simultaneously imaged
42KD (Actin)
Ctrl1,2
hDT8061,2
75 KD
50
37
25
20
15
10

B

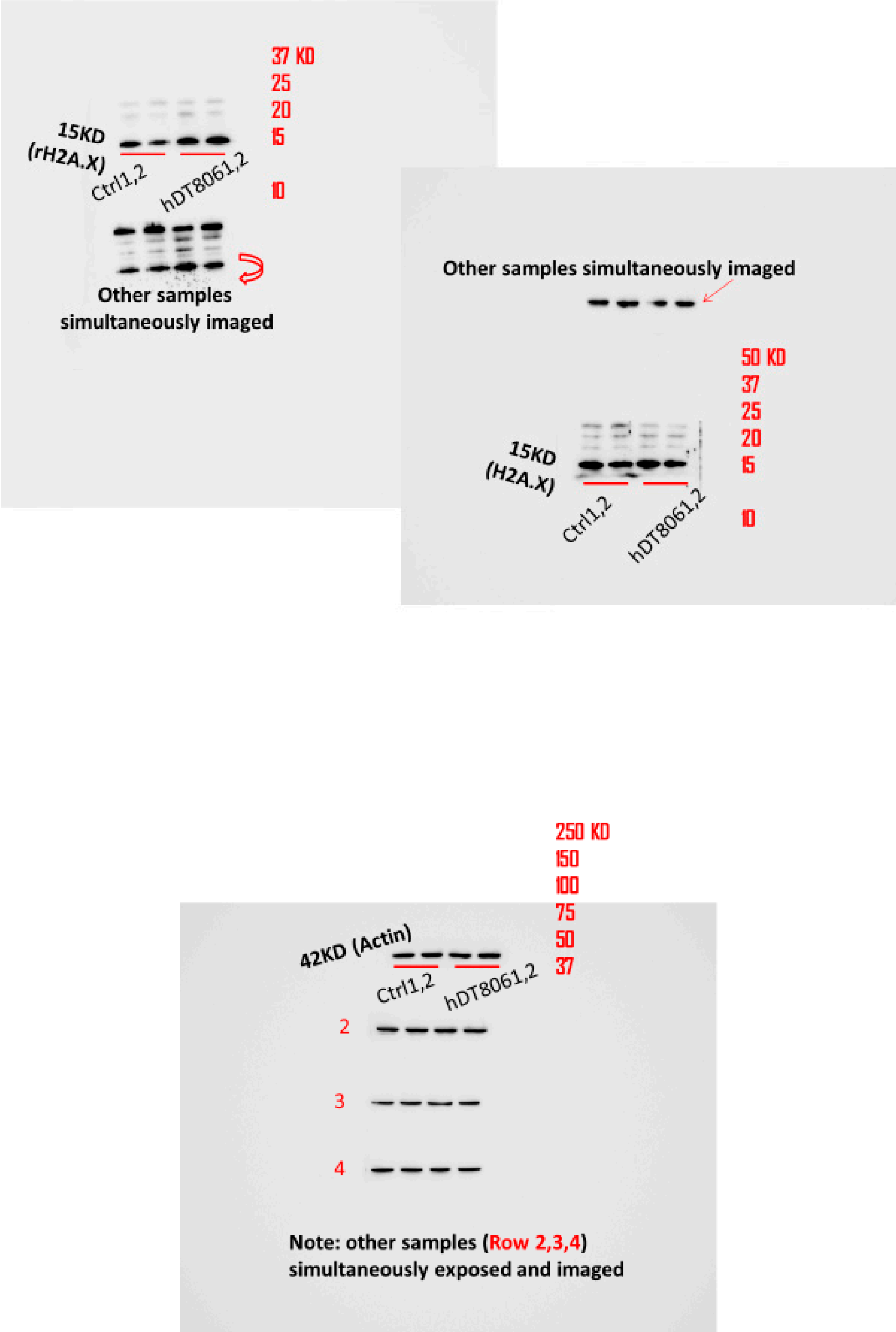

Figure S5: Analysis of hDT806 inducing transcription inhibition and DNA damage.

A

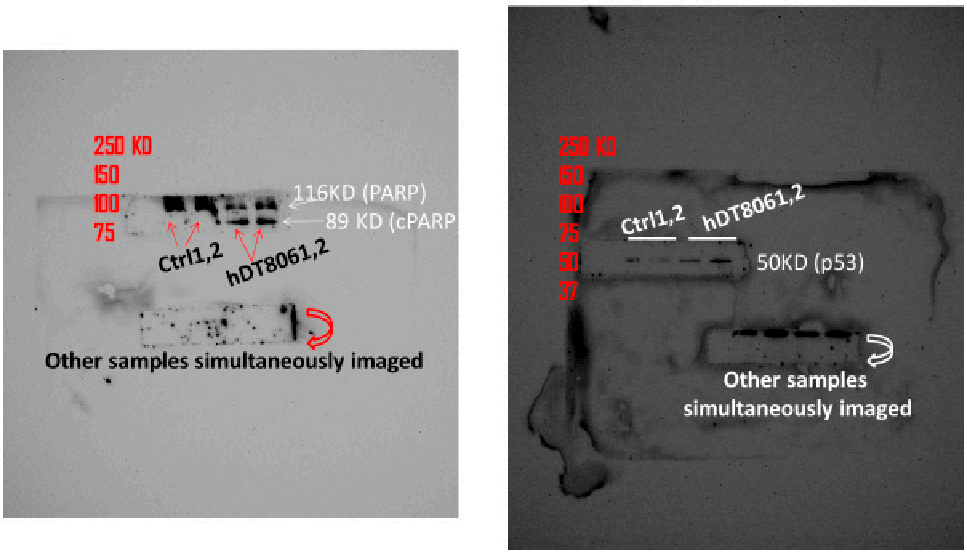
250 KD
150
100
75
116KD (PARP)
89 KD (cPARP)
Ctrl1,2
hDT8061,2
Other samples simultaneously imaged
250 KD
150
100
75
50
37
Ctrl1,2
hDT8061,2
50KD (p53)
Other samples simultaneously imaged

B

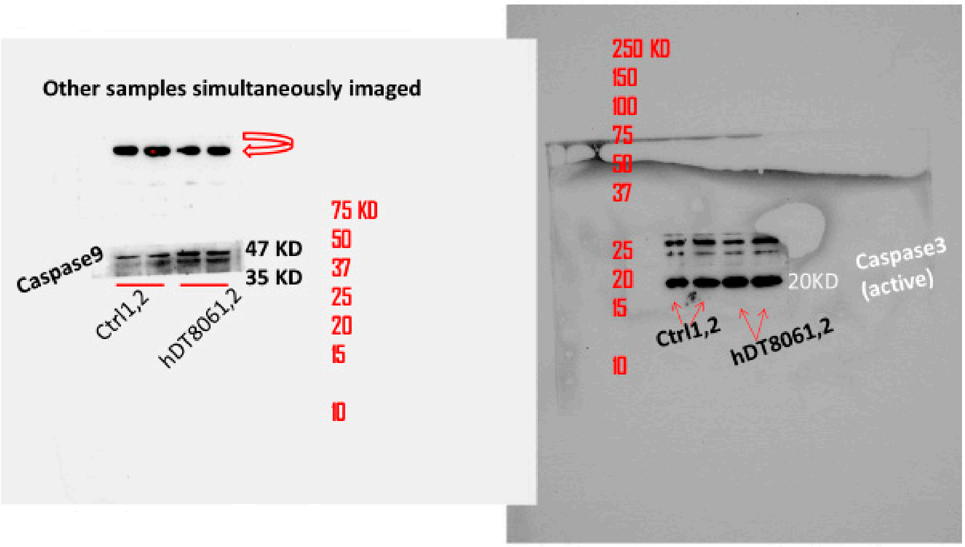
Other samples simultaneously imaged
75 KD
50
37
25
20
15
10
47 KD
35 KD
Caspase9
Ctrl1,2
hDT8061,2
250 KD
150
100
75
50
37
25
20
15
10
20KD
Caspase3 (active)
Ctrl1,2
hDT8061,2

C

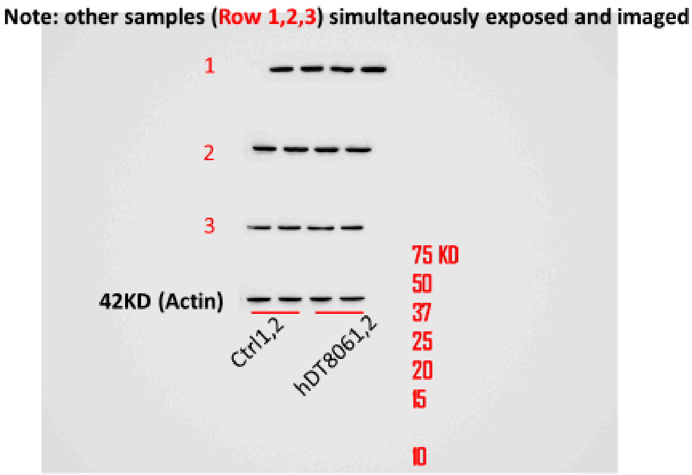

Figure S6: Ananlysis of apoptosis induced by hDT806 treatment in the JHU−029 cells.
